# Trajectories of antidepressant dispensing among privately insured transgender people in the United States

**DOI:** 10.1038/s41598-026-51351-y

**Published:** 2026-05-28

**Authors:** Theo Gabriel Beltran, Brian W. Pence, Virginia Pate, Jennifer L. Lund, Tonia C. Poteat, Kathleen C. Thomas, Shabbar I. Ranapurwala, Derrick D. Matthews, Juan M. Hincapie-Castillo

**Affiliations:** 1 Department of Epidemiology, University of North Carolina Chapel Hill, Chapel Hill, NC USA; 2 Center for Applied Transgender Studies, Chicago, IL USA; 3 Division of Healthcare in Adult Populations, Duke University School of Nursing, Durham, NC USA; 4 Division of Pharmaceutical Outcomes and Policy, University of North Carolina at Chapel Hill Eshelman School of Pharmacy, Chapel Hill, NC USA; 5 Department of Health Behavior, University of North Carolina at Chapel Hill Gillings School of Global Public Health, Chapel Hill, NC USA

**Keywords:** Computational phenotype, Transgender health, Electronic health records, Gender identity, Mental health, Depression, Anxiety, Epidemiology, Health services

## Abstract

Psychiatric medication utilization studies rarely identify transgender and gender diverse (TGD) people, masking TGD health inequities. This study assessed antidepressant prescription fill patterns and their predictors among a privately insured TGD sample with newly dispensed anti-depressants after a recent depression or anxiety diagnosis from Merative MarketScan commercial insurance claims from 2007 to 2021. We measured trajectories of antidepressants utilization over 6-months using group-based trajectory modeling (GBTM), then identified demographic and clinical predictors of medication utilization group membership. Among 2499 TGD people with depression or anxiety, we found three anti-depressant dispensing trajectory groups (short-term [21.1%], decreasing [32.3%], and long-term [46.6%]). TGD people with previous chronic co-morbidities or emergency department visits had an increased probability of shorter-term antidepressant dispensing compared to long-term dispensing. TGD people under the age of 25 and those with previous psychotherapy treatment were less likely to have shorter-term anti-depressant dispensing than those with older ages (25–64) and those without prior psychotherapy. This study provides real-world evidence of distinct anti-depressant dispensing patterns and predictive factors associated with shorter-term dispensing among TGD people. Policymakers, providers, and advocacy groups can utilize findings to inform tailored strategies to improve sustained antidepressant use among TGD people living with mental health conditions.

**Supplementary Information:**

The online version contains supplementary material available at 10.1038/s41598-026-51351-y.

## Introduction

Transgender and gender-diverse (TGD) people experience substantial mental health inequities relative to cisgender people due to discrimination and stigma toward their gender identity, leading to higher risk of depression and anxiety, with prevalence of depression reported as high as 81% and anxiety as high as 62%^1–4^. The disproportionately high burden of mental health conditions among TGD people in comparison to cisgender people is widely understood through the minority stress theory, where TGD people are subject to both distal (e.g. anti-TGD rejection and targeted legislation) and proximal (e.g. anticipated stigma against TGD people) stressors compounded with general every day stressors that lead to increased psychological distress^5–7^.

TGD people who experience gender-based discrimination, are younger than 25, and diagnosed with depression are at an increased risk of suicidality and have a prevalence of past-year suicide ideation that is eighteen times higher than the general United States (US) population^8,9^. Given the current rise in mental health conditions among the general US population, thorough research on psychiatric conditions and effective strategies for treatment and management of these conditions are urgently needed. A systematic review by Scheim et al., which characterizes disease burdens and correlates among TGD people globally, advocated for the critical need to identify individual and structural determinants of health, citing such research as an important way to develop evidence-based interventions to reduce morbidity and mortality among TGD people^10^. One intervention relevant to the mental health of TGD people is the appropriate use of pharmacological treatments. The American Psychological Association (APA) Clinical Practice Guidelines recommend antidepressants as first-line pharmacological treatment for depression^11^. The guidelines recommend at least 6–12 weeks of treatment with antidepressants to induce remission (the acute phase), followed by an additional 4–9 months (continuation phase) of maintenance treatment that may continue in order to prevent recurrence of symptoms^12,13^.

Measures of psychiatric medication dispensing are a crucial component of treatment management for people with major psychiatric disorders, as they are key indicators of effective management of chronic diseases^14–18^. One study using the Swedish Total Population Register linked with dispensing data found that after adjusting for sociodemographic factors, the prevalence of antidepressant use among TGD people was more than three times higher than the general Swedish population (aOR 3.95; 95% CI 3.62, 4.31)^19^. However, shorter-term medication dispensing fills ( referred to in past literature as non-adherence) is a common phenomenon. Non-adherence is defined by the World Health Organization (WHO) as a person’s medication taking behavior that does not correspond with recommendations from their health care provider^20^. We move away from the term “adherence” to center patient-centered language, as “adherence” suggests a patient is at fault for not taking their medication while also dichotomizing medication taking as either taking the medication or not. It is essential to understand the larger (institutional and societal) factors that support or prevent medication dispensing to reduce the stigmatization that is placed on the individual.

Importantly, medication dispensing studies often have not been able to identify TGD people, which can render invisible potential health inequities experienced by TGD populations relative to cisgender people. Literature on medication dispensing among TGD populations focuses primarily on transgender women and their dispensing of antiretroviral therapy (ART) or pre-exposure prophylaxis (PrEP), which became prioritized to urgently address the on-going HIV epidemic^21,22^. To our knowledge, there is no research that measures the duration of psychiatric medication dispensing refill patterns among TGD people.

This study aims to measure and identify longitudinal dispensing patterns of antidepressant medications used to treat depression and anxiety among TGD people using group-based trajectory modeling (GBTM). We then identify demographic and clinical predictors of antidepressant dispensing group membership to understand characteristics associated with shorter-term medication dispensing.

## Methods

Data were derived from the Merative MarketScan Commercial Claims and Encounters Database (MarketScan) from the years 2007 to 2021. MarketScan prospectively captures enrollment information and healthcare encounter data of approximately 30 to 45 million unique people each year, and represents a large portion of privately insured US patients ^23,24^. In the US, most individuals under the age of 65 have private, employer-based health insurance, which is the type of insurance captured by MarketScan. Individuals under the age of 65 can obtain insurance coverage through Medicaid (a state-run program for low-income populations or pregnant mothers and children) or through self-pay coverage; these sources are not included in the MarketScan database. As the average enrollment time was about 2.2 years, individuals can be followed through multiple years. MarketScan’s rich databases contain pertinent information on gender-affirming care (e.g., dispensed drug claims for exogenous HRT) as well as psychiatric care (e.g., psychiatric diagnoses and psychiatric medication dispensing), allowing for the evaluation of health care and mental health care dispensing among the TGD population^25–27^.

### Study population

We first identified privately insured TGD people between the ages of 13 to 64 from 2007 to 2021 using a computational phenotype (CP) consisting of both Proctor and Jasuja CP component criteria^1,28^. From the TGD population, we required an anti-depressant medication dispensing after the date they met TGD criteria. This first dispensed date was utilized as the study entry date. This antidepressant dispensing must have also occurred within 60 days of a new diagnosis of depressive disorders or anxiety (at least one inpatient or two outpatient ICD-9/10 diagnosis codes of depressive or anxiety conditions within each year). We required a washout period of 6 months prior to diagnosis where no depression or anxiety claims occurred (Appendix Table 1)^29–33^.

**Fig. 1:**
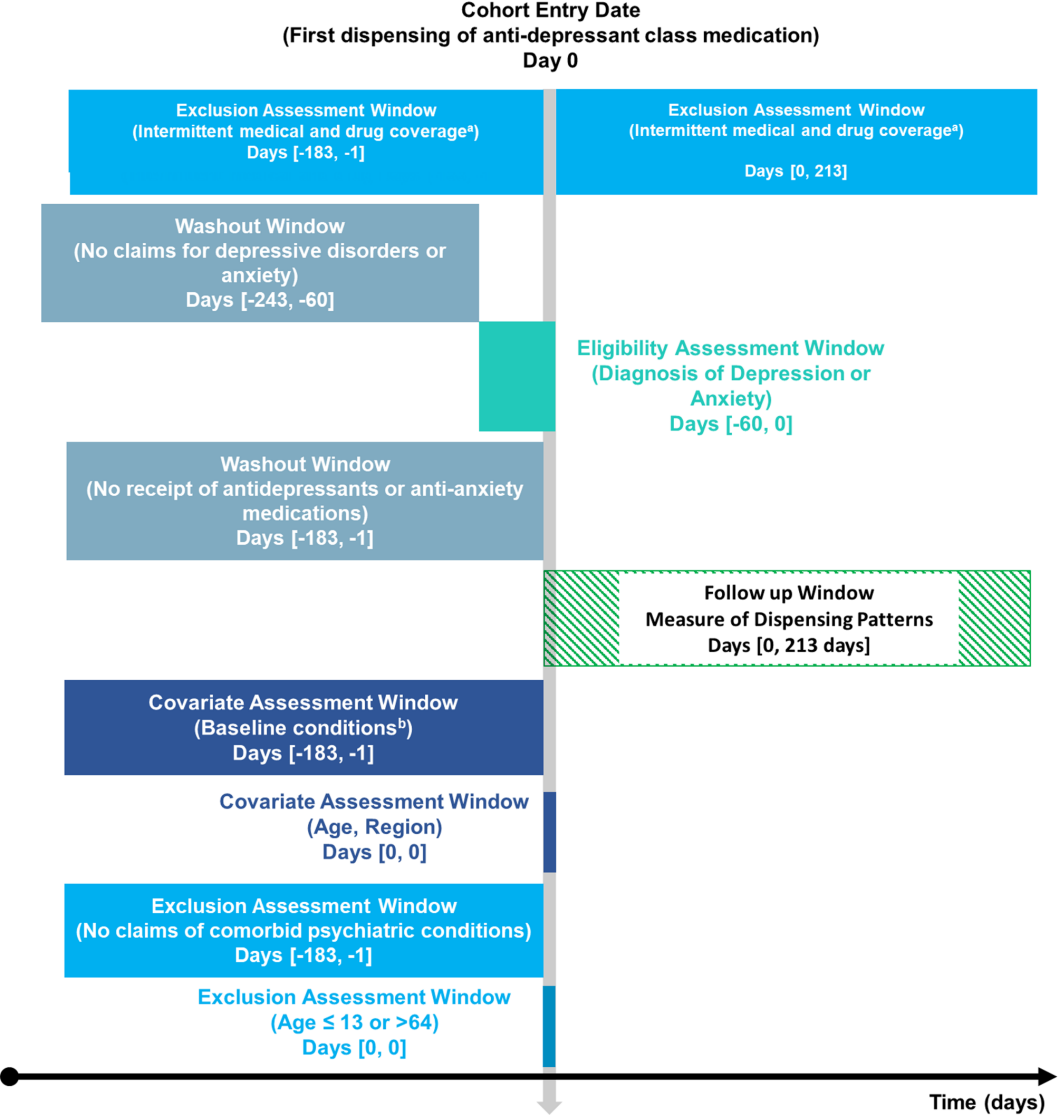
Study design. (**a**) No less than 183 days of continuous medical and drug coverage and enrollment prior to the index date, and continuous coverage (up to 213 days for 7 month follow up) and enrollment after the index date. (**b**) Baseline conditions included: Charlson Comorbidity Index, sex, age, rurality, region, number of psychiatric emergency visits, psychotherapy, and suicidality/self-harm.

TGD people must have also met the following criteria:^1^ continuous enrollment for at least 6 months prior to study entry and during the entire follow up period (7 months)^2^, “new users” of anti-depressant medication used to treat depression or anxiety, such that they had no prior dispensing of antidepressant medication within the last 6 months prior to study entry^34,35^. TGD people were excluded if they had claims for comorbid psychiatric conditions such as bipolar disorder, schizophrenia, or psychosis in the 6 months prior to index date and those hospitalized for longer than 90 days in hospitalization were excluded because we are unable to measure hospitalization medication dispensing, and people with these comorbid psychiatric conditions likely follow different guidelines^36,37^. A graphical diagram of the study design diagram is in Fig. 1.

#### Psychiatric medications

Psychiatric medications were identified through NDCs in dispensing claims for treating depressive disorders or anxiety. Antidepressants for depressive disorders or anxiety include the following therapeutic subgroups: antidepressants (selective serotonin reuptake inhibitors, serotonin and norepinephrine reuptake inhibitors, tricyclics, monoamine oxidase inhibitors, and miscellaneous). This psychotropic class of antidepressants has been utilized in past literature as they are most commonly used for first-line pharmacotherapy treatment of depression and anxiety and are listed elsewhere^38,39^.

TGD people who switch (i.e., initiate a different medication within 30 days of discontinuing a previous dispensed medication) were considered continuously exposed. Those with concomitant psychotropic dispensed medication were also included, and TGD people with more than 2 different classes of psychotropic medications were described as having polypharmacy (included in covariate section below).

Medication dispensing was measured by proportion of days covered (PDC) for incident use of psychiatric medications among TGD patients. While most pharmacoepidemiologic literature refers to the measurement of PDC for adherence assessment, we use the word “dispensing” to describe dispensing patterns in this study as not everyone with a psychiatric disorder was treated with pharmacotherapy and therefore would not be described as “non-adherent”. The monthly PDC was calculated as the number of days the drug was available (the patient had the medication “on hand” according to the days of supply recorded on each dispensing claim), divided by the thirty-day period that represents one month.

$$\:Monthly\:PDC=\left(\frac{Number\:of\:days\:drug\:was\:covered}{Thirty\:day\:time\:interval\:}\right)\times\:\:100$$

We calculated PDC in monthly intervals for purposes of fitting GBTM^40^. The PDC measure provided a conservative estimate when a medication was more prone to frequent switching or concomitant therapy as PDC covers overlaps of dispensing (which allowed the measure to get higher than 100%)^41^. For those with concurrent medications, the average dispensed-based PDC has been found to lead to less misclassification of dispensing group categorization than using only one of the medications dispensed^42^. Any oversupply for a single medication was accounted for by shifting the refill date forward after the prior refill had ended for that same medication. PDC was parameterized as a continuous variable. If a patient had an inpatient hospitalization for less than 90 days (as > = 90 day hospitalization were excluded from the cohort), we assumed those hospitalized for psychiatric reasons continued to receive antidepressant medication treatment^43^.

Given previously mentioned APA guideline recommendations, medication dispensing was followed for 6 months after the first month’s initial antidepressant date fill (7 months in total after first fill), as the first fill met study inclusion criteria^34,35,44^.

#### Potential predictor variables

We used the Charlson comorbidity index (categorized as 0, 1, 2, >=3) to measure comorbidities such as diabetes, hypertension and HIV (Appendix Table 2)^45–47^. We also included whether a patient also had previous psychotherapy claims as an additional covariate as past research has found that combined psychotherapy and pharmacotherapy is associated with increased medication dispensing^48^. We also assessed record of prior psychiatric emergency department visits and prior suicidality/self-harm before an individual’s study entry date^49^.

We assessed differences by patient demographics. For example, MarketScan claims record patient age and region/geographic location indicators. Previous research found higher dispensed antidepressant rates among adolescents aged 15–18 years compared to children and adolescents below 15 years of age^50,51^. Past literature also noted vast differences in access to antidepressants by geographic location^52^. MarketScan contains metropolitan statistical area (MSA) information that were used to measure rurality^53,54^.

### Statistical analysis

Group-based trajectory modeling (GBTM) was used to group TGD people by similar medication taking patterns over time^55^. GBTM utilizes maximum likelihood estimates to identify clusters of people within trajectories. The likelihood estimates of each individual’s repeated observations are comprised of the probability of group membership and the probability of the observed data, given their group membership. Models represent the distribution of observed data as well as the shape of each group trajectory.

GBTM assumed differences in trajectories were summarized by a finite set of different polynomial functions of time, each medication-taking group was homogenous^55^. Given the PDC was calculated as a proportion up to 100%, GBTM assumed a censored normal distribution that ranged from 0 to 1^55^. It assigned each patient a predicted probability of belonging to each latent group. The parameter estimates were useful to look at patient characteristics by group categorization.

The final trajectory model was chosen based on comparison of Bayesian information criteria (BIC) between a range of 2–7 medication-taking pattern groups (BIC closest to zero favorable), which also required a minimum 5% sample size within each latent group and the calculated average posterior probability (mean probability of individuals belonging to a specific group) of each trajectory group to be a minimum of 70%^55,56^. After selecting the optimal number of groups, we assessed the functional form of trajectories in our GBTM models from zero (constant) to the fifth order by using combinations of different forms, and looked into how functional forms changed the trajectory shape^56,57^. We also looked for reasonably narrow confidence intervals^56,58^. Two additional measurements were used to assess the best model fit. Entropy calculates classification accuracy of individuals being placed in their trajectory subgroup, with results higher than 80% as having higher accuracy. Mismatch compares the estimated probability of belonging to a subgroup compared to the proportion of individuals classified to that subgroup based on the highest posterior probability, with results closer to zero suggesting optimal fit^59^. Clinical meaningfulness and past literature were also considered to evaluate our final model choice.

We compared the group categorizations of the final model results to the calculated average PDC of each trajectory group to assess their similarity. A previous study using GBTM found differences between the maximum posterior probability and proportion distributions, and therefore heterogeneity within their moderate adherence group, which GBTM is not able to detect on its own, as it assumes homogeneity within group trajectories^56^.

We reported patient characteristics by trajectory group type, and figures depicting these dispensing groups over time will be generated using SAS (SAS Institute, Cary, NC). GBTM application was conducted using TRAJ, a GBTM package within SAS (SAS Institute, Cary, NC). Chi-square tests were used to calculate statistically significant differences (*p*<.05) of characteristics between trajectory groups.

Additionally, we conducted multinomial logistic regression to identify demographic and clinical characteristics associated with the probability of belonging to a specific latent group. The dependent variable was the probability of being a part of a latent group specified by GBTM while the independent variables were covariates such as age group, reported baseline sex, rurality, geographic region and previous emergency room (ER) visits, suicidality/self-harm, and record of co-morbidities as defined by the Charlson comorbidity index, before study entry. Stratified analysis by sex may include TGD people whose sex at enrollment is not necessarily their sex assigned at birth, as TGD people can update their sex with their health insurance. Referent groups were chosen a priori based on past literature that found these factors were associated with higher likelihood of longer term antidepressant dispensing, such as being of an older age, being female, living in a metropolitan city, having prior psychotherapy, and no previous record of suicidality/self-harm, co-morbidities, or ER visits^60,61^. The Northeast region was chosen as the referent group as previous surveys found this region had the lowest proportion of uninsured TGD people compared to other regions in the US, while the Southern region had the highest^9^.

Our study was approved by the Institutional Review Board at the University of North Carolina at Chapel Hill. All methods were performed in compliance with local regulatory and legal frameworks that govern data use.

## Results

**Fig. 2:**
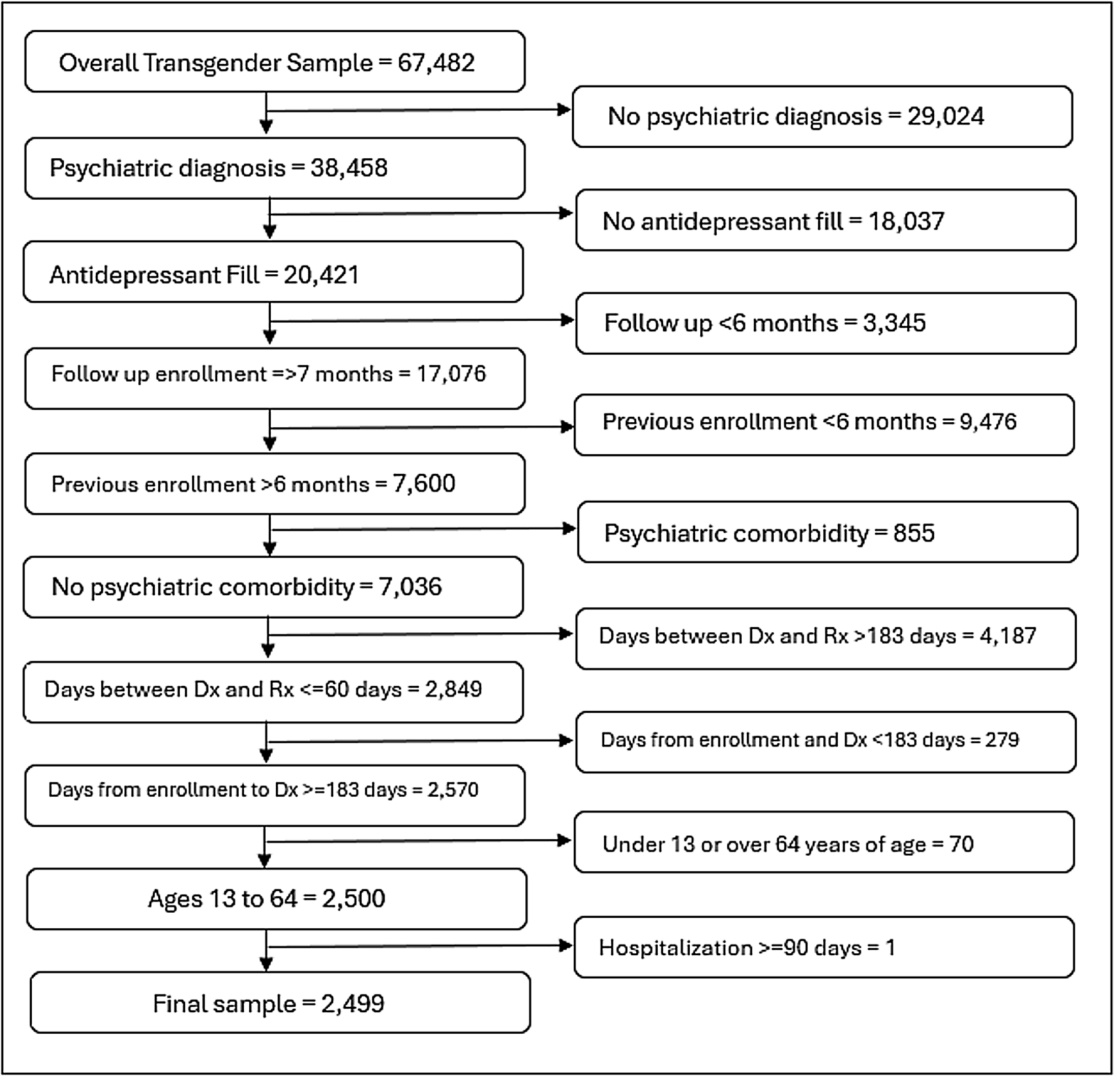
Patient selection in Merative MarketScan. Dx, Diagnosis date; Rx, First dispensing date.

Across the study period (2007–2021), our final sample contained 2499 unique patients who met inclusion criteria as TGD people enrolled in private health insurance who utilized gender-affirming care procedures, diagnosed with depression and/or anxiety, and dispensed a first-line antidepressant pharmacotherapy treatment (Fig. 2).

At baseline, TGD people were an average of 25 years of age, most were assigned a female sex at enrollment (61.5%), and received care in the Southern (37.0%) or North Central (22.3%) census regions. The mean PDC over 6 months was 46.5% [95% confidence interval (CI) (45.3, 47.7)] and the median was 49.4%. The mean PDC for TGD patients diagnosed with depression (with or without anxiety) and anxiety (with or without depression) was 47.2% (45.9, 48.5) with a median of 50.0% and 45.9.0% (44.5, 47.3) with a median of 48.9%, respectively. At baseline (prior to index date), the majority of TGD people did not receive previous psychotherapy (59.7%), 9.7% met the definition of previously recorded suicidality or self-harm during the study period, less than one-fifth met criteria for having at least one co-morbidity (19.6%), and 36.1% had a previous ER visit (Table 1). Due to large overlap of depression and anxiety diagnoses, there were no major differences between depression, anxiety, and combined groups (Supplemental Figs. S1–2). We also found no major difference when we included TGD people with co-morbid psychiatric conditions (Supplemental Fig. S3) as sensitivity analyses.

**Table 1:** Sample characteristics.

Characteristic	Total *N* = 2499 (%)	Depression, with or without anxiety, *n* = 2080 (%)	Anxiety, with or without depression *n* = 1968 (%)
Mean age (95% CI)	24.5 (24.1, 25.0)	23.7 (23.2, 24.1)	24.0 (23.5, 24.4)
Mean PDC^a^ (95% CI)	46.5% (45.3, 47.7)	47.2% (45.9, 48.5)	45.9% (44.5, 47.3)
Median PDC^a^	49.4%	50.0%	48.9%
Age category			
13–18	955 (38.2)	869 (41.8)	784 (39.8)
19–25	762 (30.5)	627 (30.1)	600 (30.5)
26–64	782 (31.3)	584 (28.1)	584 (29.7)
Sex reported at enrollment			
Male	962 (38.5)	784 (37.7)	725 (36.8)
Female	1537 (61.5)	1296 (62.3)	1243 (63.2)
Region			
Northeast	469 (18.8)	386 (18.6)	380 (19.3)
North Central	557 (22.3)	472 (22.7)	434 (22.1)
South	924 (37.0)	765 (36.8)	721 (36.6)
West	549 (22.0)	457 (22.0)	433 (22.0)
Rurality			
Yes	207 (8.3)	169 (8.1)	151 (7.7)
No	2,292 (91.7)	1911 (91.9)	1817 (92.3)
Prior psychotherapy			
Yes	1007 (40.3)	1683 (80.9)	815 (41.4)
No	1492 (59.7)	397 (19.1)	1153 (58.6)
Prior suicidal behavior			
Yes	243 (9.7)	235 (11.3)	180 (9.1)
No	2256 (90.3)	1845 (88.7)	1788 (90.9)
Prior Charlson Comorbidity Index			
>=3	31 (1.2)	25 (1.2)	23 (1.2)
2	34 (1.4)	29 (1.4)	25 (1.3)
1	247 (9.934)	204 (9.8)	204 (10.4)
0	2187 (87.5)	1821 (87.5)	1716 (87.2)
Prior psychiatric emergency visits			
Yes	902 (36.1)	757 (36,4)	711 (36.1)
No	1597 (63.9)	1323 (63.6)	1257(63.9)

N, Sample size; SD, Standard deviation; PDC, proportion of days covered; CI, confidence interval.

^a^Measured over-time during study period.

### GBTM antidepressant dispensing types

**Fig. 3:**
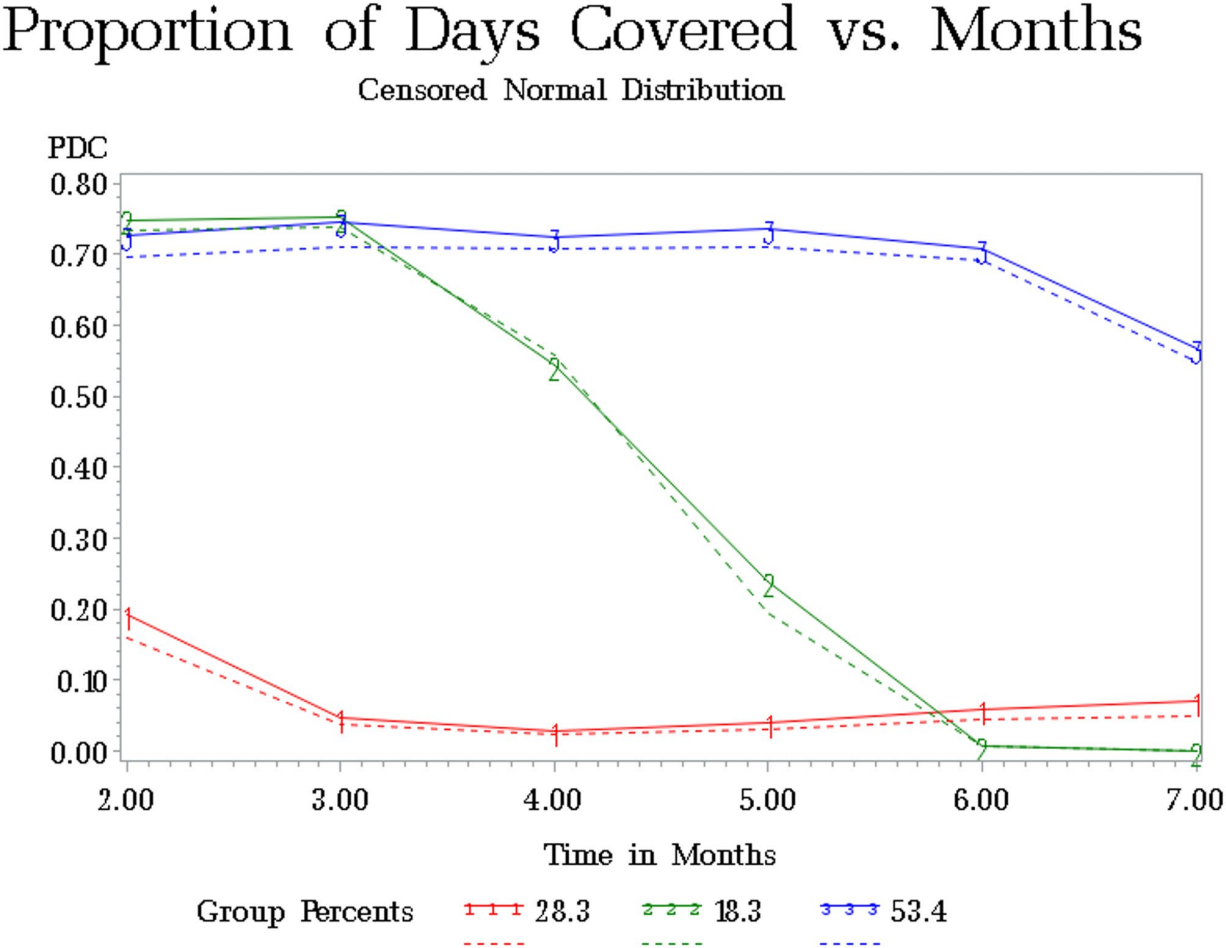
Final selected 3 group-based trajectory model.

The GBTM analysis identified three different dispensing pattern types for antidepressants (Fig. 3).

Criteria justifying our selection of the best fit model (BIC, > 5% group size, entropy, and mismatch) are displayed in Table 2.

**Table 2:** Assessment of group-based trajectory modeling results by trajectory number for model selection.

Characteristic assessed	2- Group	3-Group	4-Group	5-Group	6-Group
Size of smallest group	37.2	18.2	15.7	11.8	9.2
BIC	− 13,183.35	− 12,562.26	− 12,173.33	−11,951.10	− 11840.40
Entropy	0.926	0.907	0.886	0.885	0.849
Highest mismatch	0.12	− 0.29	1.08	− 0.99	− 1.42

We describe these three distinct medication types below as: short-term dispensing (very early discontinuation at the 2nd month, likely that the enrollee filled the medication only 1–2 times), Decreasing dispensing (gradually declining dispensing over time, dropping off after the 4th month), and long-term dispensing (stable dispensing throughout entire study period). We also provide further evaluation of the final model criteria that are sufficiently met (average posterior probability > 70%, high overlap between estimated population and sample size in each group) in Table 3.

**Table 3:** Categorizations of final 3 group model selection (without risk factors).

GBTM utilization group	Average posterior probability	Estimated population in group	Sample in group
Early discontinuation	94.4%	18.3%	18.2%
Decreasing	92.2%	28.3%	28.6%
Long-term	98.2%	53.4%	53.2%

Average posterior probability: the calculated average posterior probability among individuals belonging to a subgroup: Over 70% is sufficient. Estimated population in group: the estimated probability of belonging to a subgroup. Sample in group: the proportion of individuals classified to a subgroup based on the highest posterior probability.

The majority of TGD people were assigned to the long-term dispensing group (53.4%), followed by decreasing dispensing (28.3%), then early discontinuation (18.3%) over the included 6-month study period. We also found similar results when stratifying by psychiatric diagnosis (depressive disorders and anxiety) (Supplementary Figs. S1–S2).

Descriptive comparison results of each group by PDC and demographic characteristics are provided in Table 4.

**Table 4:** Characteristics by group trajectory subgroups from final GBTM model.

Characteristic	Utilization trajectory groups		
	Early discontinuation	Decreasing	Long-term
Proportion (%)	21.1%	32.3%	46.6%
Average PDC (%)	2.8%	32.9%	75.6%
PDC (> 80%)	0.0 (0.0)	0.0 (0.0)	465 (39.9)
PDC (< 80%)	526 (100.0)	808 (100.0)	700 (60.1)
Age category			
13–18	171 (32.5)	275 (34.0)	509 (43.7)
19–25	166 (31.6)	254 (31.4)	342 (29.4)
26–64	189 (35.9)	279 (34.5)	314 (27.0)
Sex at baseline enrollment			
Male	210 (39.9)	301 (37.3)	451 (38.7)
Female	316 (60.1)	507 (62.8)	714 (61.3)
Region			
Northeast	96 (18.3)	146 (18.1)	227 (19.5)
North Central	105 (20.0)	181 (22.4)	271 (23.3)
South	189 (35.9)	298 (36.9)	437 (37.5)
West	136 (25.9)	183 (22.7)	230 (19.7)
Rurality			
Yes	54 (10.3)	71 (8.8)	82 (7.0)
No	472 (89.7)	737 (91.2)	1083 (93.0)
Prior psychotherapy			
Yes	200 (38.0)	294 (36.4)	513 (44.0)
No	326 (62.0)	514 (63.6)	652 (56.0)
Prior suicidality/self-harm			
Yes	55 (10.5)	79 (9.8)	109 (9.4)
No	471 (89.5)	729 (90.2)	1056 (90.6)
Prior Charlson comorbidity index score			
>=3	7 (1.3)	10 (1.2)	14 (1.2)
2	7 (1.3)	17 (2.1)	10 (0.9)
1	42 (7.9)	89 (11.1)	116 (10.0)
0	477 (89.5)	688 (85.6)	1022 (88.0)
Prior psychiatric emergency visit			
Yes	100 (19.0)	105 (13.0)	147 (12.6)
No	426 (81.0)	703 (87.0)	1018 (87.4)

The average PDC was 2.8% for the short-term dispensing group, 32.9% for the decreasing dispensing group, and 75.6% for the long-term dispensing group. GBTM found that about 40% of TGD people placed in the long-term dispensing group would be defined as having “high dispensing” (> 80% dispensing) based on the binary PDC variable. Across sex and age categories, there were similar distributions among each trajectory group, although those aged 13–18 had a slightly higher proportion in the long-term dispensing group than older age groups and TGD people aged 26 or older had a slightly higher proportion in the early discontinuation group than younger age groups. Additionally, there were slightly higher proportions of TGD people in the long-term dispensing group who had prior psychotherapy but were not living in rural areas, and did not have prior suicidality or self-harm, co-morbidities, or emergency room visits than the two shorter-term dispensing groups.

### Predictive factors of shorter-term dispensing

Statistical results from the multinomial logistic regression analyses of predictive covariates of short-term dispensing and decreasing antidepressant dispensing groups in comparison to the long-term group are presented in Table 5. TGD people who lived in the Western region of the US (compared to the Northeast), had previous chronic co-morbidities, or emergency department visits were at an increased probability of belonging to shorter-term antidepressant dispensing groups. Additionally, TGD people under the age of 25 and those with previous psychotherapy treatment had a lower probability of having shorter-term dispensing of their antidepressant compared to older TGD people (over 25) and those without prior psychotherapy (Table 5).

**Table 5:** Predictive factors of the two shorter term dispensing groups compared to the long-term group of TGD people dispensed antidepressants.

	Group	
	Early discontinuation	Decreasing utilization
Variable	Relative risk	Relative risk
Sex (ref=female)	1.05 (0.85, 1.30)	0.94 (0.78, 1.13)
Age 13–18 (ref = 26 + years)	0.57 (0.44, 0.73)	0.61 (0.49, 0.76)
Age 19–25 (ref = 26 + years)	0.81 (0.62, 1.04)	0.84 (0.66, 1.05)
Southern region (ref=Northeast)	1.02 (0.76, 1.37)	1.06 (0.82, 1.37)
North central region (ref=Northeast)	0.92 (0.66, 1.27)	1.04 (0.78, 1.37)
Western region (ref=Northeast)	1.40 (1.02, 1.92)	1.24 (0.93, 1.64)
Rurality (ref = 0)	0.66 (0.46, 0.95)	0.79 (0.56, 1.09)
Psychotherapy (ref = 0)	0.78 (0.63, 0.96)	0.72 (0.60, 0.87)
Suicidality/self-harm (ref = 0)	1.13 (0.80, 1.59)	1.05 (0.77, 1.42)
Prior CCI score (ref = 0)	1.28 (0.66, 2.49)	1.65 (0.94, 2.88)
Prior ER visit (ref = 0)	1.71 (1.39, 2.12)	1.34 (1.11, 1.62)

Ref, reference; ER, emergency room; CCI, Chronic conditions index.

## Discussion

This study identified three distinct trajectories of antidepressant dispensing patterns among TGD people diagnosed with depressive disorders or anxiety who are privately insured in the US. GBTM identified three distinct dispensing types: short-term dispensing, decreasing dispensing, and long-term dispensing. These three separate groups did not align strongly with the binary PDC measure, revealing that GBTM was able to provide more detail on the antidepressant filling patterns of TGD people that would have otherwise not been reported through a PDC measure. Among the 2,499 TGD people who were dispensed a first-line antidepressant treatment for depressive disorder or anxiety, almost half (46.6%) had long-term dispensing of their antidepressant medication, suggesting about half of TGD people dispensed antidepressants met the recommended APA guidelines of 6 months^12^.

We compare our results to related literature of antidepressant dispensing among other populations. The three GBTM groups of our study are fewer than the five GBTM groups identified in a Swedish study that also measured anti-depressant dispensing over time. Their groups visually align with the medication taking patterns found in our study- though they have two additional groups with a^1^ later decline and^2^ improving increase^62^. They measured dispensing over a 24-month period, while ours was over a 6-month period, aligning with the American Psychiatric Association’s treatment guidelines^12^. A previous study using claims data to measure the antidepressant dispensing of reproductive aged women with hysterectomy procedures found six distinct trajectory groups over 18 months. However, the medication taking patterns were vastly different from our study such that some groups showed increasing dispensing, while our study showed short-term dispensing, decreasing dispensing, or long-term dispensing after a TGD person’s first antidepressant fill. A major difference between their study and ours is that their study entry occurred at time of hysterectomy rather than first antidepressant fill. As hysterectomy procedures themselves are associated with potential improvement of depressive symptoms, people may either continue or stop their antidepressant after their hysterectomy procedure. In comparison, our study entry date is the fill of an individual’s first antidepressant dispensing after a new diagnosis of a depressive or anxiety disorder^63^.

In Table 4, we contrasted the number of TGD enrollees in the three dispensing groups with the PDC binary measure to compare to prior studies. Among our study sample, less than half (40%) of TGD enrollees within the long-term dispensing group met the 80% cutoff in PDC measure as having “high” dispensing. A previous study that assessed antidepressant dispensing among people diagnosed with major depressive disorder in Marketscan found 33% of people had a PDC higher than 80% after 6 months, which is much higher than our study findings^34^. This may be potentially suggesting that TGD people have lower levels of antidepressant dispensing than the overall MarketScan sample as measured by the PDC^34^.

Our study found similar determinants of short-term dispensing as determined by previous literature, with no prior psychotherapy, older age, and record of co-morbidities and ER visits as common predictors of early antidepressant discontinuation^58^. However, our findings contrast previous studies that found living in rural areas and having a female sex were predictive factors for reduced dispensing. Our study found the opposite, as those living in rural areas were less likely to have shorter-term antidepressant dispensing and sex did not predict dispensing group. We hypothesize that patterns by sex were different as our dataset captures sex at enrollment rather than sex assigned at birth, which can be updated individually with one’s health insurance, likely leading to a mix of transgender men, women, and non-binary people in the “female” sex category. Our study also found living in a Western US region (compared to the Northeast) were predictors of shorter-term antidepressant dispensing, though these findings could not be corroborated in previous literature. It should also be noted that prior research evaluating the impact of short-term dispensing has found it to increase one’s risk for ER visits, rather than vice-versa, which our study found^64,65^. Our study did not find suicidality or self-harm to be a strong predictive factor to short or medium-term antidepressant dispensing. Previous research mainly measured the risk of suicidal behavior as an outcome among people with short-term antidepressant dispensing rather than assessment of suicidal behavior as a predictive factor to shorter term dispensing, though findings have varied^66,67^.

As TGD people with no prior psychotherapy, an older age, and prior record of co-morbidities and ER visits were at greater probability of having short-term antidepressant dispensing, providers may consider these factors if they implement interventions as structural support to lengthen antidepressant dispensing among this population. These interventions should be built into larger structural support that improves access to healthcare systems for TGD people. For example, our findings support prior suggestions by TGD health researchers that called for the increase in collaborative, comprehensive care such that TGD people have a “one-stop shop” for all of their medical care needs. A comprehensive care approach would involve collaborative integration through a team of providers as a holistic intervention to improve the mental health and well-being of TGD people. This type of integration among providers would especially provide structural support for TGD people taking multiple medically necessary medications^68,69^.

### Limitations

This study is not without limitations. We acknowledge that our method for measuring medication dispensing is a proxy, as we will not have access to data detailing whether a patient took their medication as dispensed or used the right dosage and frequency. However, this measure is endorsed by the Pharmacy Quality Alliance (PQA), utilized by the Centers for Medicare and Medicaid Services (CMS), and influences 41% of Medicare Star Ratings^70,71^. Proportion of Days Covered is known as the “foundation for enabling optimal results,” as it is the strongest available measure within claims data and critical for economic evaluations, policy decision-making, and insights for clinical outcomes of interest^72^. We also assumed that medications were interchangeable if there was a dispensing switch within 30 days, which could potentially overestimate a patient’s PDC. We also did not have medication strength or dosage information for a patient’s antidepressant, which could vary by individual. Without this information, there may be differences in medication dispensing by dosage strength that we are not able to measure in this study.

We were unable to determine if the length of antidepressant days’ supply was directed by one’s health provider, which is why we move away from describing the individual as “non-adherent” and towards discussing dispensing so as to understand larger barriers and facilitators that may influence antidepressant medication taking. Further, previous studies evaluating medication dispensing have also found early discontinuers to have similar health to long-term users, as this group may have improved symptoms where medication was no longer necessary^73^. Future research should explore medication use with data that includes measurement of symptoms and reasons for discontinuation of antidepressant use.

Additionally, this claims dataset may not capture all psychiatric-related claims as it is possible to receive psychiatric-related care outside of insurance coverage, such as psychotherapy. We also do not have information on symptom severity of depressive disorders and anxiety in claims data, which could be major time-varying factors impacting dispensing pattern trajectories. Claims databases also do not obtain patient-reported outcomes and therefore we are not able to measure side effects and the potential impact of side effects on treatment discontinuation. We also did not assess second-line treatment of antidepressants for TGD people with recurrent depression or anxiety. Future research should consider including evaluating antidepressant dispensing trajectories among TGD people using tricyclics and monoamine oxidase inhibitors antidepressants as a second-line treatment for a recurrent mental health condition.

Seasonal differences, particularly in depressive disorders, may have occurred for patients especially during winter months in the US (October to March), which could affect dispensing levels. As our measure was descriptive, we did not adjust for seasonal differences, especially as past findings found longer term exposures were more likely to cause confounding than short term exposures^74^. As we assessed dispensing patterns over 6 months, we did not anticipate this to be a major issue in our measurement.

MarketScan does not report income or racial demographic information of enrollees, which precludes our study from assessing potential differences by socio-economic status or race, which are important drivers of health inequities within the US^75^. Additionally, this claims dataset only contains claims from private insurance providers, limiting our results from being generalizable to the entire TGD patient population, although previous studies found most TGD people have private health insurance (> 50%)^76,77^. Finally, as there is no gold standard within claims data of gender identity, we cannot measure how many additional TGD people were not identified in the claims dataset who did not seek gender-affirming medical care. However, previous research using claims data applied TGD-related diagnosis codes alone and were able to obtain a sensitivity of 89% and specificity of 99%, suggesting that conservative CPs have high accuracy in identifying TGD people who receive gender-affirming care^78^.

## Conclusion

Using GBTM, we identified three distinct antidepressant dispensing trajectory groups of TGD people with depressive disorders or anxiety between 2007 and 2021. The majority of TGD people had long-term dispensing of their antidepressant over 6 months after study entry. TGD people who were over 26 years old, had evidence of co-morbidities, at least one prior ER visit, or no prior psychotherapy were at an increased risk of membership in a shorter-term antidepressant dispensing group. The use of GBTM allowed for more granular assessment of antidepressant medication taking patterns among TGD people than binary measures such as the PDC and revealed strong predictors of early discontinuation. These predictors provide information that providers and decision-makers can utilize to create interventions to improve dispensing of antidepressants for a historically marginalized population with high depressive disorders and anxiety burdens.

## Supplementary Information

Below is the link to the electronic supplementary material.

Supplementary Material

## Data Availability

The data that support the findings of this study are from Merative MarketScan and restrictions apply to the availability of these data, which were used under license for the current study, and so are not publicly available.
