# Supplementary material for: Trajectories of antidepressant dispensing among privately insured transgender people in the United States
Source: Sci Rep. 2026 May 28;16:24468. doi: 10.1038/s41598-026-51351-y (PMC13451220; doi:10.1038/s41598-026-51351-y)
Supplement: Supplementary file 1 [file 41598_2026_51351_MOESM1_ESM.docx]

**Appendices**

| **Appendix Table 1: ICD-9 and ICD-10 Codes Used to Identify Patients with Depressive and Anxiety Disorders in Claims Data**(30,31) | | |
| --- | --- | --- |
| **Psychiatric Disorder** | **ICD-9-CM** | **ICD 10-CM** |
| **Depressive Disorders** | 296.20, 296.21, 296.22, 296.23, 296.24, 296.25, 296.26, 296.30, 296.31, 296.32, 296.33, 296.34, 296.35, 296.36, 296.51, 296.52, 296.53, 296.54, 296.55, 296.56, 296.60, 296.61, 296.62, 296.63, 296.64, 296.65, 296.66, 296.89, 298.0, 300.4, 309.1, 311 (any DX on the claim) | F31.30, F31.31, F31.32, F31.4, F31.5, F31.60, F31.61, F31.62, F31.63, F31.64, F31.75, F31.76, F31.77, F31.78, F31.81, F32.0, F32.1, F32.2, F32.3, F32.4, F32.5, F32.9, F33.0, F33.1, F33.2, F33.3, F33.40, F33.41, F33.42, F33.8, F33.9, F34.1, F43.21, F43.23 (any DX on the claim) |
| **Anxiety disorders** | 293.84, 300.00, 300.01, 300.02, 300.09, 300.10, 300.20, 300.21, 300.22, 300.23, 300.29, 300.3, 300.5, 300.89, 300.9, 308.0, 308.1, 308.2, 308.3, 308.4, 308.9, 309.81, 313.0, 313.1, 313.21, 313.22, 313.3, 313.82, 313.83 (any DX on the claim) | F06.4, F40.00, F40.01, F40.02, F40.10, F40.11, F40.210, F40.218, F40.220, F40.228, F40.230, F40.231, F40.232, F40.233, F40.240, F40.241, F40.242, F40.243, F40.248, F40.290, F40.291, F40.298, F40.8, F40.9, F41.0, F41.1, F41.3, F41.8, F41.9, F42, F42.2, F42.3, F42.4, F42.8, F42.9, F43.0, F43.10, F43.11, F43.12, F44.9, F45.8, F48.8, F48.9, F93.8, F99, R45.2, R45.5, R45.6, R45.7 (any DX on the claim) |

| **Appendix Table 2. ICD-9 and ICD-10 Coding Algorithms for Charlson Comorbidities**(46–48) | | | |
| --- | --- | --- | --- |
| **Comorbidities** | **Deyo's ICD-9-CM +** | **ICD-10 *** | **Enhanced ICD-9-CM *** |
| Myocardial infarction | 410.x, 412.x | I21.x, I22.x, I25.2 | 410.x, 412.x |
| Congestive heart failure | 428.x | I09.9,I11.0, I13.0, I13.2, I25.5, I42.0,  I42.5-I42.9, I43.x, I50.x, P29.0 | 398.91, 402.01, 402.11, 402.91,  404.01, 404.03, 404.11, 404.13,  404.91, 404.93, 425.4-425.9, 428.x |
| Peripheral vascular | 443.9, 441.x, 785.4, V43.4 | I70.x, I71.x, I73.1, I73.8, I73.9, I77.1, | 093.0, 437.3, 440.x, 441.x, |
| disease | Procedure 38.48 | I79.0, I79.2, K55.1, K55.8, K55.9, | 443.1-443.9, 447.1, 557.1, |
|  |  | Z95.8, Z95.9 | 557.9, V43.4 |
| Cerebrovascular disease | 430.x-438.x | G45.x, G46.x, H34.0, I60.x-I69.x | 362.34, 430.x-438.x |
| Dementia | 290.x | F00.x-F03.x, F05.1, G30.x, G31.1 | 290.x, 294.1, 331.2 |
| Chronic pulmonary | 490.x-505.x, 506.4 | I27.8, I27.9, J40.x-J47.x, J60.x-J67.x, | 416.8, 416.9, 490.x-505.x, |
| disease |  | J68.4, J70.1, J70.3 | 506.4, 508.1, 508.8 |
| Rheumatic disease | 710.0, 710.1, 710.4, | M05.x, M06.x, M31.5, M32.x-M34.x, | 446.5, 710.0-710.4, 714.0-714.2, |
|  | 714.0-714.2, 714.81, 725.x | M35.1, M35.3, M36.0 | 714.8, 725.x |
| Peptic ulcer disease | 531.x-534.x | K25.x-K28.x | 531.x-534.x |
| Mild liver disease | 571.2, 571.4-571.6 | B18.x, K70.0-K70.3, K70.9, | 070.22, 070.23, 070.32, 070.33, |
|  |  | K71.3-K71.5, K71.7, K73.x, K74.x, | 070.44, 070.54, 070.6, 070.9, |
|  |  | K76.0, K76.2-K76.4, K76.8, K76.9, | 570.x, 571.x, 573.3, 573.4, 573.8, |
|  |  | Z94.4 | 573.9, V42.7 |
| Diabetes without chronic complication | 250.0-250.3, 250.7 | E10.0, E10.l, E10.6, E10.8, E10.9,  E11.0, E11.1, E11.6, E11.8, E11.9,  E12.0, E12.1, E12.6, E12.8, E12.9,  E13.0, E13.1, E13.6, E13.8, E13.9,  E14.0, E14.1, E14.6, E14.8, E14.9 | 250.0-250.3, 250.8, 250.9 |
| Diabetes with chronic complication | 250.4-250.6 | E10.2-E10.5, E10.7, E11.2-E11.5,  E11.7, E12.2-E12.5, E12.7,  E13.2-E13.5, E13.7, E14.2-E14.5, E14.7 | 250.4-250.7 |
| Hemiplegia or paraplegia | 344.1, 342.x | G04.1, G11.4, G80.1, G80.2, G81.x, | 334.1, 342.x, 343.x, 344.0-344.6, |
|  |  | G82.x, G83.0-G83.4, G83.9 | 344.9 |
| Renal disease | 582.x, 583-583.7, 585.x, 586.x,  588.x | I12.0, I13.1, N03.2-N03.7,  N05.2-N05.7, N18.x, N19.x, N25.0, Z49.0-Z49.2, Z94.0, Z99.2 | 403.01, 403.11, 403.91, 404.02,  404.03, 404.12, 404.13, 404.92,  404.93, 582.x, 583.0-583.7, 585.x,  586.x, 588.0, V42.0, V45.1, V56.x |
| Any malignancy, | 140.x-172.x, 174.x-195.8, | C00.x-C26.x, C30.x-C34.x, | 140.x-172.x, 174.x-195.8, |
| including lymphoma  and leukemia, except | 200.x-208.x | C37.x-C41.x, C43.x, C45.x-C58.x,  C60.x-C76.x, C81.x-C85.x, C88.x, | 200.x-208.x, 238.6 |
| malignant neoplasm of skin |  | C90.x-C97.x |  |
| Moderate or severe liver disease | 456.0-456.21, 572.2-572.8 | I85.0, I85.9, I86.4, I98.2, K70.4, K71.1, K72.1, K72.9, K76.5, K76.6,  K76.7 | 456.0-456.2, 572.2-572.8 |
| Metastatic solid tumor | 196.x-199.1 | C77.x-C80.x | 196.x-199.x |
| AIDS/HIV | 042.x-044.x | B20.x-B22.x, B24.x | 042.x-044.x |

Supplemental Figure S1. Final Selected 3 Group-Based Trajectory Model Among TGD People with Depression-Related Claims

Supplemental Figure S2. Final Selected 3 Group-Based Trajectory Model Among TGD People with Anxiety-Related Claims

Supplemental Figure S3. Sensitivity Analysis including TGD people with psychiatric co-morbidities resulting in similar 3 Group-Based Trajectory Model
